# Intensity expectation modifies gustatory evoked potentials to sweet taste: Evidence of bidirectional assimilation in early perceptual processing

**DOI:** 10.1111/psyp.13299

**Published:** 2018-11-15

**Authors:** Moon Wilton, Andrej Stancak, Timo Giesbrecht, Anna Thomas, Tim Kirkham

**Affiliations:** ^1^ Department of Psychological Sciences Eleanor Rathbone Building, University of Liverpool Liverpool L69 7ZA UK; ^2^ Unilever Research and Development Port Sunlight, Quarry Road East Bebington CH63 3JW UK

**Keywords:** assimilation, EEG, ERPs, expectation, gustation, source localization

## Abstract

Expectations can affect subjective sensory and hedonic ratings of tastes, but it is unclear whether they also shape sensory experience at a perceptual level. The neural correlates of the taste‐expectancy relationship were explored through EEG analysis. Using a trial‐by‐trial cueing paradigm, lingual delivery of 0.05 M or 0.3 M sucrose solutions was preceded by congruent or incongruent visual cues designed to promote anticipation of either a low‐sweet or high‐sweet solution. When participants were cued to expect low‐sweet, but received high‐sweet (incongruent cue), intensity ratings for high‐sweet decreased. Likewise, expectation of high‐sweet increased intensity ratings of low‐sweet solutions. Taste‐dependent, right central‐parietal gustatory ERPs were detected, with greater P1 (associated with greater right insula activation) and P2 peak amplitudes for high‐sweet tastes. Valid cue‐taste pairings led to specific reduced right‐lateralized N400 responses (associated with an attenuation in right insula activation) compared with invalid cue‐taste pairings. Finally, P1 amplitudes following invalidly cued low‐sweet tastes closely matched those generated by expected high‐sweet tastes, and P1 amplitudes for invalidly cued high‐sweet tastes resembled those generated by low‐sweet tastes. We conclude that, as well as modifying subjective ratings toward the anticipated intensity level, expectations affect cortical activity in a top‐down manner to induce bidirectional assimilation in the early perceptual processing of sweet taste and modulate N400 ERP components not previously associated with gustatory stimulation.

## 1 INTRODUCTION

Our experience of the sensory environment is determined not only by the physical characteristics of stimuli, but also by beliefs, prior knowledge, and expectations (Gibson, 1966, 1979; O'Regan & Noe, 2001; Rosch, Varela, & Thompson, 1991). Taste perception, in particular, is influenced by a variety of factors beyond primary sensory information. Characteristics such as color, texture, odor, price, and fat content have been shown to give rise to expectations that influence subsequent flavor evaluations (e.g., Cardello & MacFie, 2007; DuBose, Cardello, & Maller, 1980; Koch & Koch, 2003; Levitan, Zampini, Li, & Spence, 2008; Shankar, Levitan, Prescott, & Spence, 2009; Zampini, Wantling, Phillips, & Spence, 2008; Zellner & Durlach, 2003). The present investigation explores whether such expectations can directly affect primary taste processing, thus indicating a modulation of responses at a perceptual level.

Evidence is emerging that expectations can affect the neural processing of sensory stimuli. Generally, expected stimuli will evoke reduced brain activation compared with stimuli that are unexpected; for example, a reduction of activity in the fusiform area for expected face images (see Summerfield, Wyart, Mareike Johnen, & de Gardelle, 2011) in the auditory region for expected tones (Todorivic, van Ede, Maris, & de Lange, 2011) and reduced N400 EEG responses to expected visual (e.g., Bobes, Valdessosa, & Olivares, 1994; Proverbio & Riva, 2009), auditory (e.g., Besson & Faita, 1995; Painter & Koelsch, 2011), and olfactory (e.g., Castle, Toller, & Milligan, 2000; Kowalewski & Murphy, 2012) stimulation. However, this has been little explored in terms of gustatory stimuli.

Behaviorally, responses to taste are markedly susceptible to expectations, with studies showing that color can affect the identification of flavors (e.g., Du Bose et al., 1980), branding can promote flavor preference (e.g., McClure et al., 2004), and “healthy” labeling can reduce palatability ratings (e.g., Wardle & Solomons, 1994). It has been proposed that a central mechanism may underlie this effect, in that neural responses to a taste adapt to assimilate to the prior expectations (Braun‐LaTour & LaTour, 2005; Hoch & Ha, 1986; Lee, Frederick, & Ariely, 2006; Okamoto & Dan, 2013).

Despite a paucity of studies, there is some evidence that cortical responses to taste can be modulated by expectation (e.g., Grabenhorst, Rolls, & Bilderbeck, 2007; Nitschke et al., 2006; Plassmann, ODoherty, Shiv, & Rangel, 2008; Sarinopoulos, Dixon, Short, Davidson, & Nitschke, 2006; Woods et al., 2011). Nitschke et al. (2006) and Sarinopoulolos et al. (2006) found that when participants were presented with a cue that led them to believe that an upcoming bitter quinine taste would be less distasteful than it actually was, they reported it to be less aversive than when they received accurate information. Moreover, fMRI data showed that bitter taste activated the bilateral primary gustatory cortex (PGC) less strongly when preceded by a mildly aversive cue than by a highly aversive cue. No reliable changes in cortical activation as a function of pleasant taste or cue condition were detected, suggesting that the effects reported with quinine solutions reflect specific processing of aversive stimuli and a lack of generalization of the expectancy effect to other tastes. However, subjective ratings were obtained for pleasantness but not intensity, so it is possible that the expectancy effects were related to the perception of intensity and not directly to changes in pleasantness (Okamoto & Dan, 2013). Moreover, a study by Woods et al. (2011) has provided evidence of assimilation effects in relation to sweetness intensity. They found that a “very sweet” textual cue both enhanced subjective ratings of the intensity of a diluted orange juice drink and increased activations in the PGC. Interestingly, a complementary devaluation of ratings was not observed when an undiluted juice was preceded by a “less sweet” cue, and there was no alteration of cortical activation.

Both of the above‐mentioned studies observed cortical assimilation to expectation in the primary taste regions, suggesting an alteration of responses at a perceptual level. However, given the temporal limitations of fMRI, it is difficult to discern whether this top‐down information is modulating early sensory representations or whether this information is fed back through later, higher‐order processes. ERPs, on the other hand, enable the investigation of sequential stages of taste processing and can provide important information about whether effects take place at early perceptual stages of processing (P1, N1) or later cognitive stages (> 300 ms), with source‐localization methods allowing estimates of the regional origin of these effects.

In the following experiment, we recorded EEG activity to examine cue‐induced expectancy effects on the processing of sweet sucrose solutions. We employed a trial‐by‐trial cueing paradigm, combined with ratings of both anticipated and actual pleasantness and intensity of the taste stimuli. On‐screen cues were presented to indicate, either validly or invalidly, that there was a high probability that a subsequent taste stimulus would have either a low‐ or high‐sweet taste. We predicted that evidence of assimilation to the expected taste would be found in subjective ratings of taste stimuli, and that such effects would have correlates in the temporal components of EEG data. More specifically, if expectation influences the perceptual processing of taste, we would predict taste‐expectancy interactions on early sensory evoked ERP components originating in PGC regions. Moreover, based on the expectancy literature (e.g., Kutas & Federmeier, 2011), violations of expectations may result in increased N400 responses and PGC activations.

## 2 MATERIALS AND METHOD

### 2.1 Participants

Sixteen female participants, aged 19–31 years (*M* *±* *SD* =24.8 ± 3.9) took part in the study. Participants were in the normal to overweight range (body mass index [BMI] range 19.5–27.9; *M* = 23.0 ± 2.5). All participants were prescreened to ensure they were nonsmokers, nondiabetic, had no food allergies or intolerances or taste disorders, and were not taking medications or suffering illnesses that could interfere with their gustatory or olfactory perception. All participants gave informed consent, and the study was conducted in accordance with the Code of Ethics of the World Medical Association (Declaration of Helsinki) and was approved by the University of Liverpool Ethics Committee.

### 2.2 Design

A 2 × 2 within‐subject design was employed. The independent variables were taste (low‐sweet, high‐sweet) and expectation (valid cue, invalid cue). The dependent variables were ERP amplitude and current densities at ERP latencies.

### 2.3 Taste stimuli

A low (0.05 M; low‐sweet) and a high concentration of sucrose (0.3 M; high‐sweet) were selected for the study. Both tastes were presented at room temperature (23**°**C) using a computer‐controlled gustometer ensuring no differences in thermal or mechanical stimulation. Initial ratings taken prior to the experiment indicated that the high‐sweet taste was evaluated as more intense (*M* *±* *SD* = 46.2 ± 20.1) than the low‐sweet taste (10.8 ± 6.39; *t*(15) = 6.41, *p* *<* 0.001) and more pleasant (17.4 ± 22.7) than the low‐sweet taste (5.3 ± 12.3; *t*(15) = 2.09, *p* *=* 0.05).

### 2.4 Measurements

Before and during the experiment, each taste was rated for pleasantness and intensity using the Labelled Affective Magnitude scale (LAM; Schutz & Cardello, 2001) and the generalized Labelled Magnitude Scale (gLMS; Bartoshuk et al., 2004), respectively. These category‐ratio scales comprise 100‐mm lines with quasilogarithmically spaced ticks and semantic labels. Participants are required to decide which term most closely describes the taste's pleasantness (LAM) or strength (gLMS) and then to refine the rating by placing a mark between that descriptor and the next most appropriate label. For instance, if the participant feels that the sensation is a little stronger than moderate, the mark should be placed on the line in between the *moderate* and *strong* semantic labels.

Additionally, to account for any effects of hunger (e.g., Plihal, Haenschel, Hachl, Born, & Pietrowsky, 2001; Stockburger, Weike, Hamm, & Schupp, 2008), appetite was measured prior to the EEG study using a six‐part visual analogue scale (0–100 mm) measuring hunger, fullness, desire to eat, satisfaction, nausea, and thirst (Flint, Raben, Blundell, & Astrup, 2000).

### 2.5 Stimulus presentation

The taste stimuli were presented using a computer‐controlled gustometer. This comprised electronically controlled diaphragmatic pumps (KNF STEPDOS FEM03.18RC, Verder, Vleuten, The Netherlands), which delivered solutions via separate tubing to a common manifold with an inline check valve to prevent cross‐contamination. The pumps were operated using PsychoPy open‐source software (Peirce, 2007), which also interfaced with a monitor to provide instructions to the participants. The taste solutions were administered to the center of the tongue via 1.6‐mm internal diameter Teflon tubing, clamped to a headrest. Each 1‐ml taste sample was administered over 2 s at a flow rate of 30 ml/min. Technical measurements prior to the study established the rise time to be <0.02 s from the serial port signal being returned to the software.

It is important to mention here that the flow of liquid through thin Teflon tubing generates a buildup of static electricity (electrification) that, as well as being a safety concern, results in substantial noise within the EEG data. This problem was rectified by employing a grounding mechanism so that any accumulated charge could be discharged. This comprised a copper wire inserted into an unused manifold channel and attached by a length of insulated copper wire to the Faraday cage.

Prior to the administration of the taste solution, participants were presented with either a blue or yellow fixation cross on a monitor, to indicate that there was a high probability that the next taste sample would be either low‐sweet (yellow cue) or high‐sweet (blue cue). The participant then rated the expected pleasantness and intensity of the predicted taste using onscreen LAM and gLMS scales. Participants were then instructed to wait while the experimenter monitored a video link and electromyography (EMG) data, initiating taste delivery when no movement or swallowing motions were evident. Participants were required to hold the taste in their mouth for 3 s while remaining still, before rating the taste for actual intensity and pleasantness. Each tasting was followed by a 4‐s (2 ml) distilled water rinse. The ratings and waiting period allowed for an interstimulus interval (ISI) of ~30 s, thus controlling for habituation and adaptation (Evans, Kobal, Lorig, & Prah, 1993). The order of the taste samples was randomized. Overall, both taste stimuli were repeated 50 times over the course of 100 trials, separated into four blocks of 25 trials. Participants were instructed that the cues would correctly predict the taste 70% of the time, and thus were invalid for 30% of the trials.

### 2.6 Procedure

All participants began the testing procedure between 9:00 and 10:00, or between 12.30 and 13:00, and were required to eat their normal breakfast or lunch prior to testing, to ensure that there were no confounding effects of hunger on the EEG data (e.g., Plihal et al., 2001; Stockburger et al., 2008). Participants completed the appetite questionnaires and then tasted and rated a 10‐ml sample of each test solution for their intensity and pleasantness using the LAM and gLMS. Next, they rinsed their mouth with room temperature water until they could no longer taste the last solution. The EEG equipment was fitted to the participant, who was then seated in the experimental chamber. Earplugs were fitted to ensure that there were no acoustic cues to the gustometer operation. Participants each completed four practice trials, comprising two high‐sweet, validly cued trials and two low‐sweet, validly cued trials, ensuring that they associated the tastes with the appropriate colored predictive cues. Participants then completed the experimental trials, with stimuli delivered and LAM and gLMS ratings taken as described above. Overall, the experiment took approximately 1 hr to complete.

### 2.7 Electrophysiological measures: ERP and sLORETA

The data were recorded using a BioSemi ActiveTwo amplifier system (BioSemi BV, Amsterdam, The Netherlands), with 64 scalp electrodes arranged according to the International 10–20 system (Oostenveld & Praamstra, 2001) and placed in an elastic cap. Common mode sense (CMS) and driven right leg (DRL) electrodes were used as a reference and ground, respectively. The EEG was continuously recorded at 512 Hz with a band‐pass filter of 0.001–100 Hz. Two external EMG electrodes were placed over the masseter muscles to detect swallowing movements, and were sampled at 512 Hz. The EMG data were visually inspected to initiate trials in the absence of movement (see above) and were not analyzed further.

The EEG data were analyzed offline using EEGLAB (Delorme & Makeig, 2009) and sLORETA (Pascual‐Marqui, 2002) toolboxes in combination with custom MATLAB scripts. Trials in which the gustometer failed to operate correctly were excluded from analysis. Each participant's recording was low‐pass filtered at 30 Hz and then downsampled to 128 Hz to reduce file size. The continuous data were then segmented into −200 ms to 1,500 ms epochs relative to the time‐locked event that was triggered at the onset of the taste stimuli. The 200‐ms baseline was selected in line with gustatory ERP investigations (Ohla, Hudry, & le Coutre, 2009; Ohla, Toepel, le Coutre, & Hudry, 2010).

Bad channels, identified through visual inspection and kurtosis (threshold =5), were removed and interpolated (Delorme & Makeig, 2009), and the data were average‐referenced to all electrodes. An independent component analysis (ICA) was used to identify and extract ocular and other muscular artifacts (Jung et al., 2000). An average (± *SD*) of 9.2 ± 3.4 noise components were removed using ICA. There were 36.1 ± 5.6 high‐sweet taste trials and 37.7 ± 3.7 low‐sweet trials retained for each participant following the preprocessing procedures. There were no significant differences between the quantity of trials retained for each taste condition (*p* = 0.849).

For standardized low‐resolution brain electromagnetic tomography (sLORETA) analysis, electrode coordinates were determined from the 64 electrode locations, using the original recording montage, and a transformation matrix created. The averaged waveforms were converted and saved into sLOR values for each condition and participant. Computations were made in a head model (Fuchs, Kastner, Wagner, Hawes, & Ebersole, 2002), using the MNI152 template (Mazziotta et al., 2001), with the three‐dimensional solution space restricted to cortical gray matter. The intracerebral volume is partitioned in to 6,239 voxels with 5 mm spatial resolution. Anatomical labels are reported as Brodmann areas using an appropriate correction from MNI to Talairach space (Brett, Christoff, Cusack, & Lancaster, 2002). Thus, sLORETA images represent the electrical activity at each voxel in neuroanatomic Talairach space (Talairach & Tournoux, 1988) as the squared standardized magnitude of the estimated current density.

### 2.8 Statistical analysis

We performed two types of analysis: the standard time domain averaging technique to examine ERPs and sLORETA to examine the origin of the ERP effect. To evaluate mean ERP differences between taste and expectancy conditions, the EEG data were exported to MATLAB R2012b (The MathWorks, Inc., USA) and analyzed using one‐way repeated measures analysis of variance (ANOVA), for each condition at each electrode and each time point in the range from −200 to 1,500 ms. This data‐driven analysis allows for the exploration of the entire epoch for potential differences between conditions or groups; allowing clusters of signals and intervals of interest to be identified. A 95% confidence interval and a permutation technique with 500 random permutations was employed (Maris & Oostenveld, 2007) in the EEGLAB v. 9 program package (https://sccn.ucsd.edu/eeglab/) to control for the Type I error associated with multiple comparisons. Briefly, the permutation technique randomly partitions trials into subsets and calculates a test statistic on the random partition. This is repeated a set number of times (in our case, 500), and a histogram of test statistics is created. The *p* value reflects the proportion of random partitions that results in a larger test statistic than the one observed; if this is <0.05, we can conclude significant differences between the experimental conditions. For a more detailed description of this technique, see Maris and Oostenveld (2007).

Subsequently, neighboring electrodes showing statistically significant differences between conditions and, with a time window > 30 ms in duration, were combined into clusters and averaged. Once identified, the ERP clusters were evaluated for each participant and then subjected to a series of within‐subject ANOVAs with the factors Taste (low‐sweet, high‐sweet), Expectancy (valid cue, invalid cue) and Taste × Expectancy using SPSS v20 (IBM, 2011).

When significant ERP components were identified, sLORETA was used to compute the cortical three‐dimensional distribution of the current density at each significant latency, the maximum current density being taken as the source of the particular component. We calculated sLORETA images for each ERP in the time frame −200–1,500 ms poststimulus. sLORETAs for each source were obtained for each participant and subjected to a series of within‐subject ANOVAs with the factors Taste, Expectancy, and Taste × Expectancy.

Post hoc analyses using pairwise comparisons and Bonferroni corrections were conducted for each EEG analysis when significant main effects occurred. Greenhouse‐Geisser corrections were applied when statistical assumptions were not met. Where multiple significant effects occurred, results were collated to show the smallest mean difference, greatest standard error, and greatest *p* values, respectively (*MD*s>, *SE*s <, *p*s <). Effect sizes (ES) represent the partial η^2 ^value.

A series of analysis of covariance (ANCOVA) tests were performed to assess whether any of the data covaried with BMI or appetite ratings. No interactions of BMI (*p*s > 0.29) or appetite (*ps* > 0.08) with behavioral or EEG data were identified, and these factors are not discussed further.

Parametric assessments were performed on behavioral data as the taste responses were found to be normally distributed (skewness range = 0.133–0.965, *SE* = 0.564).

## 3 RESULTS

### 3.1 Behavioral analysis

#### 3.1.1 Intensity ratings

**Figure 1:**
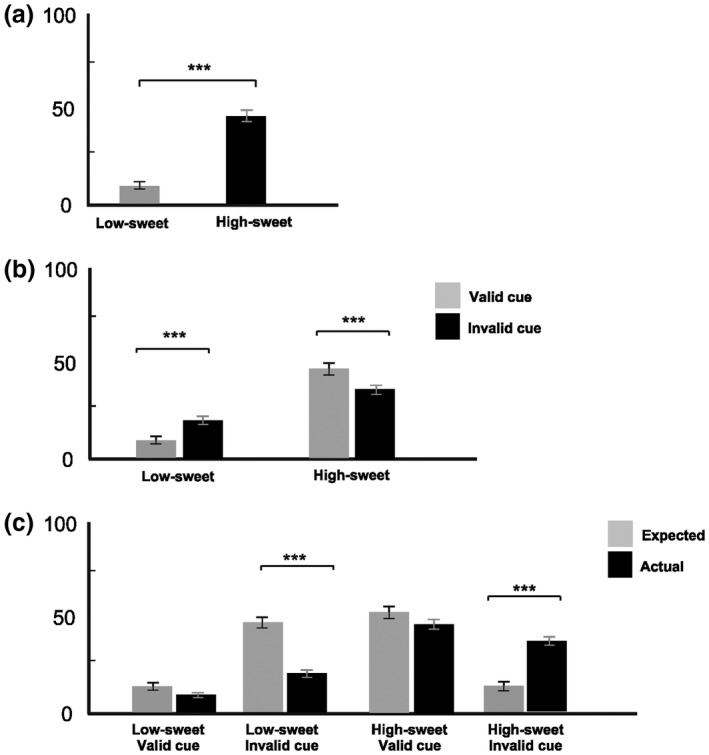
Intensity ratings for low‐sweet and high‐sweet tastes (a) prior to testing, (b) separately for validly and invalidly cued conditions, and (c) under validly and invalidly cued conditions taken before (expected) and after (actual) tasting. All values are the mean ± *SE*. *p *<* 0.05; **p *<* 0.01; ***p *<* 0.001

We first examined the influence of Taste and Expectancy on mean intensity ratings taken after tasting (Figure 1a,b) using a 2 × 2 repeated measures ANOVA. We then compared predicted and actual taste intensity ratings in validly and invalidly cued trials for each taste (Figure 1c) using 2 × 2 × 2 repeated measures ANOVA.

There was a significant effect of Taste on intensity ratings, with high‐sweet rated as more intense than low‐sweet taste, *F*(1, 15) = 94.58, *p* < 0.001, ES = 0.86 (Figure 1a). Importantly, a significant interaction between Taste and Expectancy on intensity ratings was apparent, *F*(1, 15) = 41,72 *p* < 0.001, ES = 0.74 (Figure 1b). Invalidly cued low‐sweet tastes (on trials when high‐sweet tastes were expected) were rated as more intense than validly cued low‐sweet tastes. In contrast, invalidly cued high‐sweet tastes (on trials when low‐sweet tastes were cued) were rated as less intense than validly cued high‐sweet taste.

Confirming the successful manipulation of expectancy, we also observed marked contrasts between predicted and actual intensity ratings for each taste when there was a mismatch between expectation and actual sweetness level, *F*(1, 15) = 52.68, *p* < 0.001, ES = 0.79 (Figure 1c). When expectations were met, ratings of predicted intensity were much more similar to actual intensity ratings.

#### 3.1.2 Pleasantness ratings

We first examined the effect of Taste and Expectancy on pleasantness ratings using a 2 × 2 repeated measures ANOVA. We then compared predicted and actual taste intensity ratings in validly and invalidly cued trials for each taste using 2 × 2 × 2 repeated measures ANOVA.

Analysis of pleasantness ratings revealed no significant effects of Taste (*p* *=* 0.329) or Expectancy condition (*p* *=* 0.321), and no interaction (*p* *=* 0.41). There was also no difference between predicted and actual ratings of taste pleasantness when there was a mismatch between expectation and actual sweetness level (*p* *=* 0.489). As noted above, despite the initially higher pleasantness reported for the high‐sweet solution prior to the expectancy testing, both tastes were judged similarly for pleasantness over the course of the experiment.

### 3.2 ERP and sLORETA analysis

**Figure 2:**
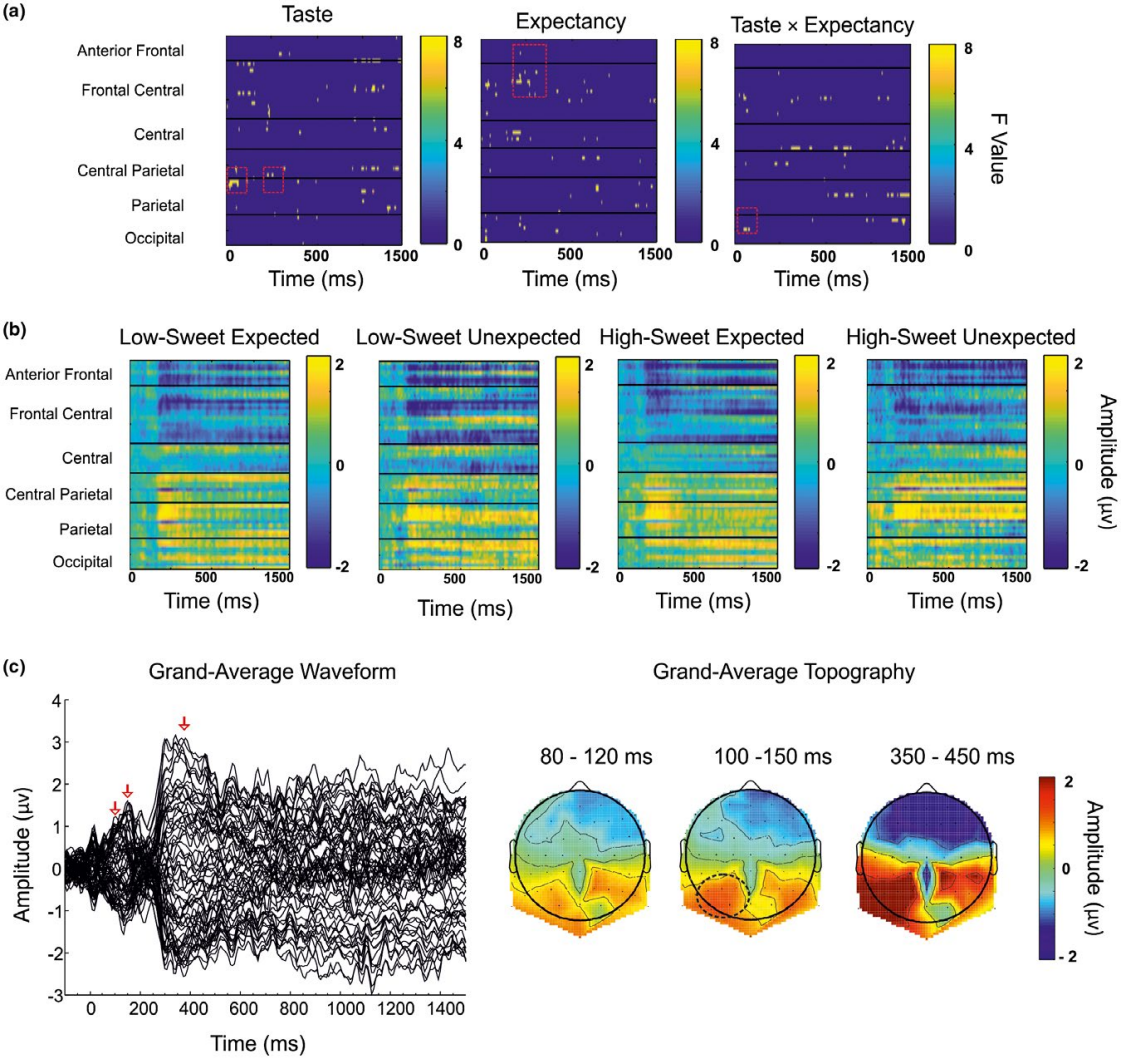
(a) Heat map plots of *F* values for each independent variable with electrodes arranged left to right, front to back. Each line represents an electrode, with bar charts representing *F* value. (b) Heat map plots of amplitude (μV) for each condition. Each line represents an electrode, with bar charts representing amplitude (μV). (c) Butterfly plot of grand‐averaged waveforms at all electrode sites. (d) Scalp topographies of mean amplitude over the 80–100 ms, 100–150 ms, and 350–450 ms epochs

**Figure 3:**
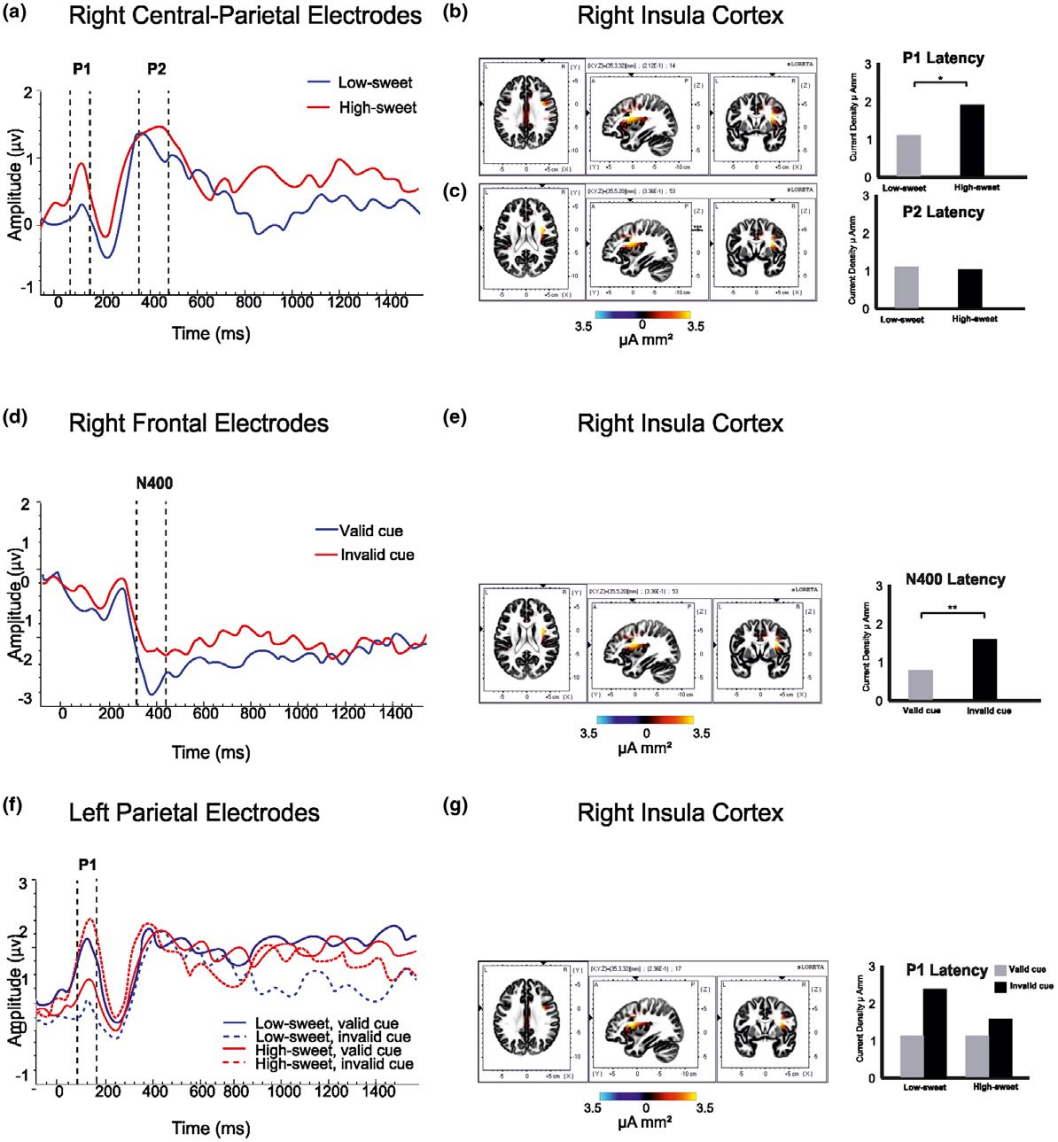
Temporal ERP amplitude plots (μV), with vertical dashed lines indicating intervals with significant main effects of condition; sLORETA images displaying maximum current density at each ERP latency for the grand mean results (color bars representing current density, μA mm^2^) and bar charts showing the current density for each condition. (a) Significant ERP effects of taste in the left frontal region (CP2, CP4, CP6, P2, P6) at P1 (80–120 ms) and P2 (350–450 ms). Maximum current density at P1 (b) and P2 taste latencies (c), located in the right insula cortex, with bar charts showing the respective mean current densities and latencies for each taste condition. (d) N400 ERP effects of Expectancy (valid/invalid cue) in right frontal region (AF4, AF8, F2, F4). (e) Maximum current density at the N400 expectancy latency located in the right insula cortex with bar charts showing the respective mean current densities and latencies for each. (f) Significant ERP amplitude effects of Taste × Expectancy in the left parietal region (P9, PO3, PO7) at P1 (100–150 ms). (g) sLORETA image showing the maximum current density at the P1 Taste × Expectancy latency located in the right insula cortex with bar chart showing the mean current density at this location and latency for each taste and expectancy condition. *p *<* 0.05; **p *<* 0.01; ***p *<* 0.001

As can be seen from Figure 2, the one‐way ANOVAs identified distinct electrode clusters and time windows that displayed significant effects for each of the independent variables (a) and across conditions (b). The grand‐averaged ERP was calculated and confirmed the time windows (c) and regions (d) showing peak activity. The data were analyzed, and distinct ERPs were observed, with peak effects detected across the −200–1,500 ms epoch (Figure 3). Effects that appeared from the one‐way ANOVA but did not show significance upon further post hoc assessments have not been discussed (i.e., those seen at later latencies in Figure 2a). The significant ERP and current density effects for Taste, Expectancy, and Taste × Expectancy interactions are summarized below.

#### 3.2.1 Taste

Effects of taste on ERP data occurred in right‐central parietal region (CP2, CP4, CP6, P2, P6). A significant effect emerged at the P1 component (80–120 ms), *F*(1, 15) = 20.15, *p* < 0.001, ES = 0.57. As can be seen from Figure 3a, high‐sweet tastes evoked a greater P1 peak (*M* *±* *SE* = 1.09 ± 0.24 µV) than low‐sweet tastes (0.78 ± 0.17 µV). sLORETA analysis indicated that the effect at P1, originated from the right insula cortex (Talairach coordinates [TAL], *x* = 33, *y* = 4, *z* = 21; Figure 3b). There was a significant effect of Taste on current density, *F*(1, 15) = 5.57, *p* = 0.023, ES =0.27, which was significantly increased in responses to high‐sweet (1.89 ± 0.35 µA mm^2^) compared with the low‐sweet tastes (1.22 ± 0.26 µA mm^2^).

A similar effect was also detected for the P2 ERP component (350–450 ms), *F*(1, 15) = 4.64, *p* = 0.048, ES = 0.57, when the high‐sweet taste again evoked greater peak amplitude (1.66 ± 0.42 µV) than the low‐sweet taste (1.20 ± 0.36 µV). The ERP plot (Figure 3a) shows this separation continuing between 650–1,000 ms, although this did not achieve significance (*p* *=* 0.121). sLORETA analysis indicated that, at P2, the greatest activations were again estimated to originate from the right insula cortex (TAL, *x* = 33, *y* = 4, *z* = 21; Figure 3c), but no differences in current densities for the taste conditions emerged (*p* = 0.633).

#### 3.2.2 Expectancy

ERP effects of Expectancy (valid/invalid cue) occurred in the right frontal region (AF4, AF8, F2, F4). As can be observed from Figure 3d, the valid and invalidly cued conditions evoked similar amplitudes at the P1 peak (150–250 ms; *p* = 0.66). However, an N400 component (350–450 ms) was also evident for Expectancy, with evidence of differential effects of congruent and incongruent trials. Validly cued tastes elicited a reduced peak (−1.78 ± 0.37 µV) compared with those that were invalidly cued (−2.39 ± 0.28 µV), *F*(1, 15) = 4.93, *p* = 0.042, ES = 0.25. These differences were not reliably sustained for the remainder of the epoch. (*p*s > 0.188).

sLORETA analysis indicated that the N400 effect originated from the right insula cortex (TAL, *x* = 33, *y* = 4, *z* = 21; Figure 3e), where there was a significant effect of Expectancy on current density, *F*(1, 15) = 8.86, *p* = 0.009, ES = 0.37. Invalidly cued tastes elicited a greater current density (1.55 ± 0.46 µA mm^2^) than validly cued tastes (0.75 ± 0.21 µA mm^2^).

#### 3.2.3 Taste × Expectancy interactions

Taste × Expectancy interactions on ERP data were observed in the left parietal region (P9, PO3, PO7; *F*(1, 15) = 5.29, *p* = 0.036, ES = 0.26). As illustrated in Figure 3f, at the P1 component (100–150 ms) validly cued low‐sweet tastes evoked a greater P1 amplitude (1.08 ± 0.35 µV) than when they were invalidly cued (0.14 ± 0.35 µV). In contrast, invalidly cued high‐sweet tastes evoked an increased amplitude (2.28 ± 0.45 µV) compared with validly cued high‐sweet (0.95 ± 0.45 µV). In effect, a low‐sweet taste preceded by a high‐sweet cue generated an ERP that more closely matched the response to a correctly anticipated high‐sweet taste; a high‐sweet taste preceded by a low‐sweet cue induced a response similar to that after a correctly anticipated low‐sweet taste. sLORETA analysis indicated that at the P1 latency the greatest current densities were observed in the right insula cortex (TAL, *x* = 33, *y* = 4, *z* = 21; Figure 3g), but the Taste × Expectancy interaction on current densities failed to reach significance (*p* *=* 0.08).

## 4 DISCUSSION

We investigated the influence of expectations on the temporal and regional processing of tastes, evaluating EEG data across all electrodes and time points relative to the onset of high‐ and low‐sweet tastes under validly or invalidly cued conditions. Behaviorally, expectancy influenced subjective intensity ratings, with evident assimilation of evaluations toward expected levels on incongruent trials. These subjective effects were reflected in the EEG analysis in relation to ERPs that were consistently source‐localized to the right insula, within the PGC. Gustatory ERPs were observed, with intensity‐dependent P1 and P2 differences evident within the right central parietal area. Taste‐Expectancy interactions occurred in early (P1) components. Moreover, incongruent trials resulted in N400 amplitude differences not previously observed for gustatory stimulation. These novel findings suggest that expectancies affect the processing of gustatory stimuli at early perceptual and attentional levels. These data support conclusions drawn from studies investigating other sensory modalities (e.g., Kutas & Federmeier, 2011) and gustatory fMRI investigations indicating expectation‐induced assimilation in early taste perception (Nitschke et al., 2006; Sarinopoulos et al., 2006; Woods et al., 2011).

### 4.1 Taste

High‐sweet tastes were subjectively rated as more intense than low‐sweet tastes. However, although the high‐sweet solution was initially rated as more pleasant than low‐sweet, there were no effects of taste pleasantness throughout the duration of the EEG study. It is possible that participants may have habituated to the pleasantness of the taste over the course of the experiment, despite long ISIs designed to avoid this. It is also possible that sucrose concentration cannot be assumed to be a simple proxy measure for relative liking. Nonetheless, that the P1 and P2 amplitude of the gERP were shown to be increased for the high‐sweet compared with the low‐sweet tastes supports previous data indicating taste intensity‐dependent amplitude shifts for these gERP components (Hummel, Genow, & Landis, 2010; Ohla et al., 2010). Moreover, we were able to discern that these gERPs were localized to the right insula cortex, with greater current densities for high‐sweet tastes. These findings corroborate earlier reports of intensity‐dependent changes in primary gustatory regions (e.g., Ohla et al., 2010).

### 4.2 Expectancy

In the right frontal region, an enhanced N400 was seen in response to invalidly cued stimuli. The N400 latency was source‐localized to the right insula region in the PGC where activations to invalidly cued stimuli were greater compared with those that were validly cued. The N400 ERP typically has a centroparietal topography and is associated with unexpected outcomes, particularly in language processing (Kutas & Federmeier, 2011). However, research into a range of sensory inputs has demonstrated topographic differences in the N400 distribution. For instance, incongruencies in facial images produce N400 effects across visual occipital areas (e.g., Olivares, Iglesias, & Bobes, 1999), while odor–object mismatches produce a frontal N400 (Grigor, Van Toller, Behan, & Richardson, 1999). Thus, the N400 can be regarded as a functional entity that varies topographically with different sources of sensory input (Kutas & Federmeier, 2011). For the first time, this phenomenon can be now extended to incongruency in primary gustatory processing and, although novel, it is not surprising that we find the source of this component to originate from PGC regions.

### 4.3 Taste × Expectancy interactions

Intensity ratings for high‐sweet tastes decreased when participants were expecting a low‐sweet taste, while intensity ratings for low‐sweet tastes increased when a high‐sweet taste was expected. Importantly, taste‐expectancy interactions were evident in early ERP processing, discernible in the left parietal region. The P1 amplitudes for invalidly cued low‐sweet tastes were decreased to a level similar to those generated by expected high‐sweet tastes. Additionally, P1 amplitudes for invalidly cued high‐sweet taste increased to a level similar to those evoked by validly cued low‐sweet tastes. These data indicate that assimilation to expectancy not only occurs at a behavioral level (e.g., DuBose et al., 1980; Levitan et al., 2008; Shankar et al., 2009; Zampini et al., 2008), but for the first time show that this effect may be mediated at an early perceptual level of neural processing.

Moreover, compared to fMRI reports, our data confirm that assimilation is not limited to aversive tastes (e.g., Nitschke et al., 2006; Sarinopoulos et al., 2006) and can be bidirectional (Woods et al., 2011), with a low‐sweet cue resulting in a modified neural response to high‐sweet tastes and a high‐sweet cue resulting in an altered cortical response to low‐sweet tastes. The current results, therefore, extend the findings reported in fMRI investigations and show the first evidence that intensity perception and concomitant neural processing tend to shift toward responses that would be engendered were the actual taste stimulus to match expectation, regardless of the direction of the expectation.

### 4.4 Limitations

Although discriminable intensity ratings of the sweet solutions were evident throughout the experiment, pleasantness evaluations did not differ consistently, despite clear differences in initial ratings and the measures taken to reduce habituation and adaptation. Consequently, the current data cannot provide any clear inference about the influence of, or changes to, the hedonic value of the stimuli in relation to stimulus congruency. Our strategy of collecting data in a single session was designed to reduce EEG noise associated with multiple test days and potential variation in electrode sites. The issue of stabilized hedonic responses should be accounted for in any future EEG study measuring responses to repeated taste stimuli in order to specifically address manipulation of expectations of palatability. Nevertheless, the present study has reliably determined that expectancy modifies perceived intensity of intrinsically pleasant, sweet stimuli and evokes cue‐dependent changes in cortical activation. Moreover, since our expectancy cues were presented solely in terms of relative sweetness, it is likely that they may not have directly affected anticipated or actual palatability evaluations, so that our findings specifically reflect alterations in the processing of intensity (Okamoto & Dan, 2013).

There is also a question of whether gLMS data should be treated as being measured on a continuous scale. Magnitude estimations and the gLMS are assumed to generate ratio level data. However, there has been evidence of “clustering” around the semantic labels—suggestive of categorical behavior (Hayes, Allen, & Bennett, 2013). While this gLMS compression is undesirable, it has been noted that this is accompanied by decreased variance, which allows for better distinction between responses to tastes (Lawless, Sinopoli, & Chapman, 2010) and reduces ceiling effects associated with the alternative visual analog scaling. In line with the majority of research published in this area, we maintain the use of the gLMS as a continuous scale. We found our data to be normally distributed, and thus the statistical model was not compromised. Other studies may prefer to use visual analog scaling in order to avoid such clustering. However, this method sacrifices the semantic information about the magnitude of the responses gained from the gLMS, which can be valuable in studies similar to the current investigation.

Although not necessarily a specific limitation of the current study, another factor that may be considered in future studies is the nature of cortical activation related to the anticipatory cues themselves. Evidence from the fMRI studies described earlier suggests that regional changes while viewing a cue may determine whether, to what extent, and in which direction the cues influence the perception and evaluation of taste stimuli (Okamoto & Dan, 2013; Sarinopoulus et al., 2006). While the current investigation measured responses to the tastes following the cue to examine the modification of taste processing following anticipation, future investigations may benefit from examining cortical activity at the time of the cue to determine if this may have some predictive value on the following responses.

Lastly, we note that the topography across the epochs (Figure 2c) differs from those observed in some gustatory EEG studies. The P1 gustatory ERP is generally frontally located (e.g., Ohla et al., 2010), reported to peak later (e.g., 130–150 ms; for a review, see Ohla, Busch, & Lundström, 2012), and can show different latencies for different stimulus concentrations (e.g., Hummel et al., 2010), although these results are not consistent across studies (e.g., Kobayakawa, Saito, Gotow, & Ogawa, 2008; Saito et al., 1998). It is possible that the early posterior positivity observed here may be explained by the overlap with gustatory and lingual somatosensory potentials. This may be accounted for in future by providing a tasteless solution with which to compare ERPs or by habituating the tongue to the lingual touch response (e.g., Crouzet, Busch, & Ohla, 2015; Iannilli, Singh, Schuster, Gerber, & Hummel, 2012; Kobayakawa et al., 1996; Kobayakawa, Ogawa, Kaneda, Ayabe‐Kanamura, & Saito, 1999; Onoda, Kobayakawa, Ikeda, Saito, & Kida, 2005; Singh, 2011; Singh, Hummel, Gerber, Landis, & Iannilli, 2015), although care must be taken to avoid lengthy testing durations and frequent masseter movements, which can cause unwanted artifacts within the data.

### 4.5 Conclusions

In sum, sweet taste intensity‐dependent P1 and source‐localized PGC intensity effects were observed, further highlighting the significance of early ERPs and the PGC in taste intensity processing (e.g., Hummel et al., 2010; Ohla et al., 2010). We also demonstrated that the N400 ERP component, previously observed for incongruencies within other sensory modalities, can be detected in response to unexpected taste stimuli and is also generated from within PGC areas. Importantly, our data demonstrate that prior expectations can not only modify subjective intensity ratings of taste, but can also affect early sensory representations measured using the fine temporal resolution enabled by analysis of EEG responses.

These data strengthen and extend conclusions drawn from fMRI studies that report taste‐expectation interactions in primary gustatory cortices (Nitschke et al., 2006; Sarinopoulos et al., 2006; Woods et al., 2011) and show that early sensory representations can be modified by top‐down information. Moreover, the current study extends the analysis of expectation specifically to the processing of sweet taste intensity and provides the first demonstration of clear bidirectional effects of expectancy cues on cortical activation by pleasant, sweet tastants.

Taken together, these findings show that taste intensity perception and concomitant neural processing tend to assimilate toward responses that would be engendered were the actual taste stimulus to match expectation. This phenomenon has important wider implications for understanding the role of extrinsic cues involved in food choice and experience. For instance, such assimilation effects may explain why a “healthy” or “low‐fat” food label can result in decreased selection and palatability ratings compared with the same product without this information (e.g., Bowen, Tomoyasu, Anderson Carney, & Kristal, 1992; Koster, Beckers, & Houben, 1987; Light, Heymann, & Holt, 1992; Wardle & Solomons, 1994) and thus has implications for developing healthier food alternatives that are free from negative hedonic expectations (Davidenko et al., 2015).
